# Same Abbreviated Injury Scale Values May Be Associated with Different Risks to Mortality in Trauma Patients: A Cross-Sectional Retrospective Study Based on the Trauma Registry System in a Level I Trauma Center

**DOI:** 10.3390/ijerph14121552

**Published:** 2017-12-11

**Authors:** Cheng-Shyuan Rau, Shao-Chun Wu, Pao-Jen Kuo, Yi-Chun Chen, Peng-Chen Chien, Hsiao-Yun Hsieh, Ching-Hua Hsieh

**Affiliations:** 1 Department of Neurosurgery, Kaohsiung Chang Gung Memorial Hospital and Chang Gung University College of Medicine, Kaohsiung 88301, Taiwan; ersh2127@cloud.cgmh.org.tw; 2 Department of Anesthesiology, Kaohsiung Chang Gung Memorial Hospital and Chang Gung University College of Medicine, Kaohsiung 88301, Taiwan; shaochunwu@gmail.com; 3 Department of Plastic Surgery, Kaohsiung Chang Gung Memorial Hospital and Chang Gung University College of Medicine, Kaohsiung 88301, Taiwan; bow110470@gmail.com (P.-J.K.); libe320@yahoo.com.tw (Y.-C.C.); VENU_CHIEN@hotmail.com (P.-C.C.); sylvia19870714@hotmail.com (H.-Y.H.)

**Keywords:** injury severity, mortality, trauma, Abbreviated Injury Scale (AIS), Injury Severity Score (ISS)

## Abstract

The Abbreviated Injury Scale (AIS) measures injury severity of a trauma patient with a numeric method for ranking anatomy-based specific injuries. The summation of the squares of the three most severe injuries in the AIS of six predefined body regions comprises the Injury Severity Score (ISS). It assumes that the mortality of a given AIS value is similar across all body regions. However, such an assumption is less explored in the literature. In this study, we aimed to compare the mortality rates of the patients with the same AIS value in different injured body regions in a level I trauma center. Hospitalized adult trauma patients with isolated serious to critical injury (AIS of 3 to 5) between 1 January 2009, and 31 December 2016, from the Trauma Registry System in a level I trauma center were grouped according to the injured body regions (including, the head/neck, thorax, abdomen, or extremities) and were exclusively compared according to their AIS stratum. Categorical data were compared using the two-sided Fisher exact or Pearson chi-square tests. ANOVA with Games-Howell post hoc test was performed to assess the differences in continuous data of the patients with injury in different body regions. The primary outcome of the study was in-hospital mortality. The adjusted odds ratios (AORs) were estimated using a stepwise selection of a multivariable regression model adjusted by controlling the confounding variables such as sex, age, comorbidities, and ISS. Survival curves were estimated with the Kaplan–Meier approach with a corresponding log-rank test. The patients with AIS of 5 for abdomen injury and those with AIS of 3 for extremity injury had a significantly lower odds of adjusted mortality (adjusted odds ratio (AOR) 0.1, 95% confidence interval (CI) 0.01–0.39, *p* = 0.004 and AOR 0.3, 95% CI 0.15–0.51, *p* < 0.001, respectively) than that of the patients with head/neck injury. However, the patients with AIS of 4 for extremity injury demonstrated significantly higher odds of adjusted mortality (AOR 8.4, 95% CI 2.84–25.07, *p* < 0.001) than the patients with head/neck injury. This study found that the risks to mortality in the patients with a given AIS value of serious to critical injury in different injured body regions were not the same, even after controlling for confounding variables such as sex, age, comorbidities, and ISS.

## 1. Background

The Abbreviated Injury Scale (AIS) was developed in 1971 to measures injury severity of a trauma patient with a numeric method for ranking anatomy-based specific injuries [1]. The AIS assesses the severity of the anatomical injury on a six-point ordinal scale ranging from minor (1), moderate (2), serious (3), severe (4), critical (5), to un-survivable injury (6), and served as a fundamental base of many severity scoring systems. For example, the Injury Severity Score (ISS), created by Baker et al. in 1974, is the summation of the squares of the three most severe injuries in the AIS of six predefined body regions [2]; the New Injury Severity Score (NISS) introduced by Osler et al. in 1997, is the summation of the squares of the three most severe injuries, regardless of the body regions injured [3]; and the Exponential Injury Severity Score (EISS), created in 2014 by modifying the AIS system, is mathematically expressed as EISS = 3^A−2^ + 3^B−2^ + 3^C−2^, where A, B, and C are the three most severe AIS codes [4,5].

As the scores of these different systems in injury severity assessment are based solely on the AIS, their final scores will be greatly influenced by the accuracy of AIS in determining individual anatomical injury severity. The AIS scores are created according to many dimensions of the injury, including impacted energy, extent of organ damage, threat to life, permanent impairment, and period of management [1]. The mortality of a given AIS value is assumed to be similar across all body regions. Under such condition, for example, it supposes the mortality rate will be similar for a patient with an AIS of 5 in the head/neck region and in the thorax, as well as for a patient with an AIS of 4 in the head/neck region and in the abdomen. However, such an assumption is less explored in the literature. Therefore, we aimed to compare the mortality rates of the patients with the same AIS value in different injured body regions in a level I trauma center. Considering the mortality rates are relatively low for the patients with AIS of 1 or 2 and extremely high for the patients with AIS of 6, only the patients with isolated serious to critical injury (AIS of 3 to 5) in only one body region were included in this study. The primary hypothesis of this study was that the patients with a given AIS value in different injured body regions would have a similar risk to mortality.

## 2. Methods

### 2.1. Study Design

**Figure 1:**
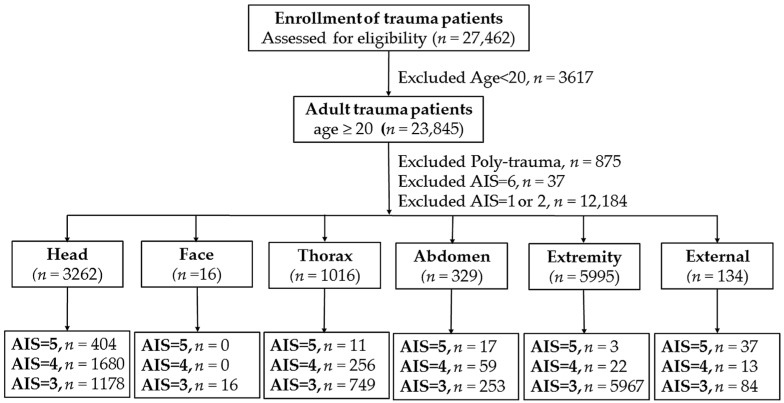
Flow chart of the studied patients with injuries to different body regions in AIS of 3–5.

This study was approved by the Institutional Review Board (IRB) of Kaohsiung Chang Gung Memorial Hospital (reference number 201701603B0), a level I regional trauma center providing care to trauma patients, primarily from southern Taiwan [6,7]. According to the regulations of the IRB, the informed consent was waived. This retrospective study reviewed all hospitalized adult patients (*n* = 23,845) registered in the Trauma Registry System from 1 January 2009 to 31 December 2016. To avoid the confounding effect of injuries to other body regions, for those patients with AIS = 3 or 4 injuries to body region, polytrauma patients [8] with additional AIS scores of ≥3 points in any other region of their body were excluded. Furthermore, because the number of patients with isolated AIS = 5 injury to one body region was too few for a powerful statistical analysis, therefore additional group of patients with AIS = 5 injury to either body region was included and the polytrauma patients were not excluded from the analysis especially for this studied group of patients. In addition, patients with a fatal (AIS = 6, *n* = 37) or mild (AIS = 1 or 2, *n* = 12,184) injuries were also excluded from the study. Thus, the patients included in this study were defined as having isolated serious to critical injury (AIS of 3 to 5) in only one body region (Figure 1), and were allocated into groups with injury to the head/neck (*n* = 3262), face (*n* = 16), thorax (*n* = 1016), abdomen (*n* = 329), extremity (*n* = 5995), and external (*n* = 134) regions. Patients with isolated injury to the face region were excluded from further analysis owing to the limited number of individuals (*n* = 16) in this group. Similarly, some patients with isolated injury to the external injury (*n* = 134) who also had burn injuries were excluded because the latter injuries induces a different presentation and outcome from trauma patients. Finally, the study population included adult patients with serious to critical injury to one body region in the head/neck, thorax, abdomen, or extremity, and each was exclusively divided into three groups: AIS of 3, 4, or 5 (Figure 1). Detailed patient information retrieved from the Trauma Registry System included the following: sex; age; vital signs, including temperature (°C), heart rate (beats/min), respiratory rate (times/min), systolic blood pressure (mmHg), upon arrival at emergency department; comorbidities, such as diabetes mellitus (DM), hypertension (HTN), coronary artery disease (CAD), congestive heart failure, cerebrovascular accident (CVA), and end-stage renal disease (ESRD); trauma mechanism; ISS, data was presented as the median with interquartile range; hospital length of stay; the rates of admission into the ICU; and in-hospital mortality.

### 2.2. Statistical Analysis

The patients were stratified into groups with an AIS of 3, 4, or 5. The injury characteristics and outcomes of the patients with injury in different body regions (thorax, abdomen, or extremity) were compared to those with injury in the head/neck region, who comprised the largest portion of the studied population. We used the SPSS (version 20.0, IBM Corp., Armonk, NY, USA) for all statistical analyses. The categorical data was compared with Pearson chi-square or Two-sided Fisher exact tests with presented odds ratios (ORs) and 95% confidence intervals (CIs). The homogeneity of variance of continuous data was estimated using Levene’s test, and then ANOVA with Games-Howell post hoc test was used to assess the differences between the patients with injury in head/neck region and those with injury in regions of thorax, abdomen, or extremity. The continuous data were presented as mean ± standard deviation. For those patients with isolated AIS = 3 or 4 injury to one body region, the adjusted odds ratios (AORs) were estimated using a stepwise selection of a multivariable regression model adjusted by controlling the confounding variables such as sex, age, comorbidities, mechanisms, and ISS, with the 95% CI of this AOR calculated. For those patients with combined AIS = 5 injury, potential confounding factor as AIS ≥ 3 to other body region was used as additional variable in the calculation of AORs in the multivariable regression model. The primary outcome of the study was mortality of the patients in the hospital. Survival curves were estimated with the Kaplan-Meier approach with a corresponding log-rank test. *p*-values < 0.05 were defined as statistically significant.

## 3. Results

### 3.1. Injury Characteristics and Outcomes of Patients with AIS of 5

Among these groups of patients with injury of AIS of 5, those with an extremity injury were not analyzed because of the limited number of individuals (*n* = 3) in this group. As shown in Table 1, no significant difference in sex, age, comorbidities, and injury mechanism was noted between the patients with thorax injury and head/neck injury. Patients with abdomen injury were younger, presented with lower odds of HTN, and had a higher incidence of injury with most of these patients being the motor vehicle drivers, but with a lower incidence of injury from a fall than those with abdomen injury. There was no significant difference in ISS between the patients with thorax or abdomen injury and those with head/neck injury. As shown in Table 2, in the group of patients with combined AIS = 5 injury to the regions of the head/neck (*n* = 1680), thorax (*n* = 256), abdomen (*n* = 59), and extremity (*n* = 22), the patients with thorax injury were significantly younger, presented with lower odds of HTN, and with a higher incidence of injury as a motor vehicle passenger than those with head/neck injury (Table 3); the patients with abdomen injury were significantly younger, presented with lower odds of HTN, and had a higher incidence of injury as the motor vehicle drivers, but a lower incidence of injury from a fall than those with head/neck injury; the patients with extremity injury were still too few for a powerful analysis (Table 3). Regarding to ISS, those patients with injury to abdomen (*p* = 0009), but not to thorax (*p* = 0.090), were significantly severely injured than those with injury to head/neck. Compared to the patients with head/neck injury, those with AIS = 5 injury to the thorax and abdomen had significantly 3.0- and 2.8-fold of associated AIS ≥ 3 injuries to other body regions. Those with injury to the thorax and did not had significantly different odds of mortality than the patients with head/neck injury, regardless under the adjustment of potential confounders (AOR, 0.4; 95% CI, 0.14–1.25; *p* = 0.118) or under log-rank test (*p* = 0.219). In contrast, those with injury to the abdomen had a significantly lower odds of mortality than the patients with head/neck injury (OR, 0.04; 95% CI, 0.01–0.32; *p* < 0.001), even after adjustment (AOR, 0.1; 95% CI, 0.01–0.39; *p* = 0.004) or under log-rank test (*p* < 0.001).

### 3.2. Injury Characteristics and Outcomes of Patients with AIS of 4

In the patients with AIS of 4, a significant male predominance in the patients with thorax injury was noted when compared to that of patients with head/neck injury, but no significant difference was noted when comparing patients with abdomen or extremity injury to those with head/neck injury (Table 4 and Table 5). The patients with thorax and abdomen injury, but not extremity injury, were significantly younger than those with head/neck injury. Regarding the comorbidities, patients with thorax injury presented with lower odds of HTN, CVA, and ESRD, while those with abdomen injury presented with lower odds of DM and HTN, and the patients with extremity injury presented with lower odds of HTN when compared to the patients with head/neck injury. In the patients with thorax injury, majority of the patients were drivers or passengers in a motor vehicle accident, hence fewer patients in this group sustained a bicycle accident or fall injury as compared to those with head/neck injury. Similarly, in the patients with abdomen injury, as majority of the patients were drivers in a motor vehicle accident as well as victims who were struck by a vehicle, there were fewer patients who sustained a fall injury as compared to the patients with head/neck injury. In the patients with extremity injury, there was a higher rate of fall injury than in the patients with head/neck injury. The patients with thorax injury had a significantly higher ISS than the patients with head/neck injury, but there was no significant difference of ISS between the patients with abdomen or extremity injury and the patients with head/neck injury. No significant differences in mortality were noted between the patients with injuries to the thorax and abdomen vs. head/neck. The adjusted odds of mortality was higher in the patients with extremity injury (AOR, 8.4; 95% CI, 2.84–25.07; *p* < 0.001) when compared to those with head/neck injury. No significant differences were noted in adjusted mortality between the patients with injuries to the thorax and head/neck (AOR, 0.3; 95% CI, 0.09–1.01; *p* = 0.051) as well as between the patients with injuries to the abdomen and head/neck (AOR, 2.1; 95% CI, 0.64–6.80; *p* = 0.220). Regarding the mortality rate of the patients with extremity injury or abdomen vs. head/neck, the log-rank tests of Kaplan–Meier survival analysis remained the similar conclusion with those driven from adjusted mortality. However the log-rank test revealed there was significant difference of mortality rates between the patients with injuries to the thorax than to the head/neck.

### 3.3. Injury Characteristics and Outcomes of Patients with AIS of 3

In the patients with AIS of 3, a significant male predominance in patients with thorax injury and a female predominance in the patients with extremity injury than in the patients with head/neck injury were noted, but no significant difference was noted in the patients with abdomen injury than in patients with head/neck injury (Table 6 and Table 7). Compared to the patients with head/neck injury, the patients with abdomen injury were significantly younger, but patients with extremity injury were significantly older. Regarding the comorbidities, the patients with thorax injury presented with lower odds of CVA, those with abdomen injury presented with lower odds of HTN, and those with extremity injury presented with higher odds of DM, HTN, CAD, CVA, and ESRD than the patients with head/neck injury. Compared to the patients with head/neck injury, the patients with thorax injury had a higher rate of patients as a driver in a motor vehicle accident and sustained a struck injury, but a lower rate of patients sustained a bicycle accident and was injured as a pedestrian; the patients with abdomen injury had a higher rate of patients as a driver in a motor vehicle accident, who was injured in a fall accident, and as a victim in a struck injury but a lower rate of patients in a bicycle accident and in a fall injury; patients with extremity injury had sustained a lower rates of injures as a driver or passenger in a motor vehicle accident, as a motorcycle driver, as a bicyclist, and as a pedestrian but had a higher rate of fall injury. The patients with thorax injury had a significantly higher ISS than the patients with head/neck injury, but the patients with abdomen or extremity injury had a significantly lower ISS than the patients with head/neck injury. Compared to the patients with head/neck injury, the patients with extremity injury had significantly lower odds of mortality (OR, 0.5; 95% CI, 0.28–0.83; *p* = 0.009), but no significant difference of mortality was noted between the patients with injury to the thorax or abdomen and the patients with injury to the head/neck. The adjusted odds of mortality showed similar results as the patients with extremity injury had significantly lower odds of adjusted mortality (AOR, 0.3; 95% CI, 0.15–0.51; *p* < 0.001) than the patients with head/neck injury and there was no significant difference of the adjusted mortality between the patients with injury to the thorax or abdomen and the patients with injury to the head/neck. The log-rank tests of Kaplan–Meier survival analysis remained the similar conclusion with those driven from adjusted mortality of the patients with injury to thorax, abdomen, or extremity vs. head/neck.

### 3.4. Summary of the Odds of Adjusted Mortality in the Patients

**Figure 2:**
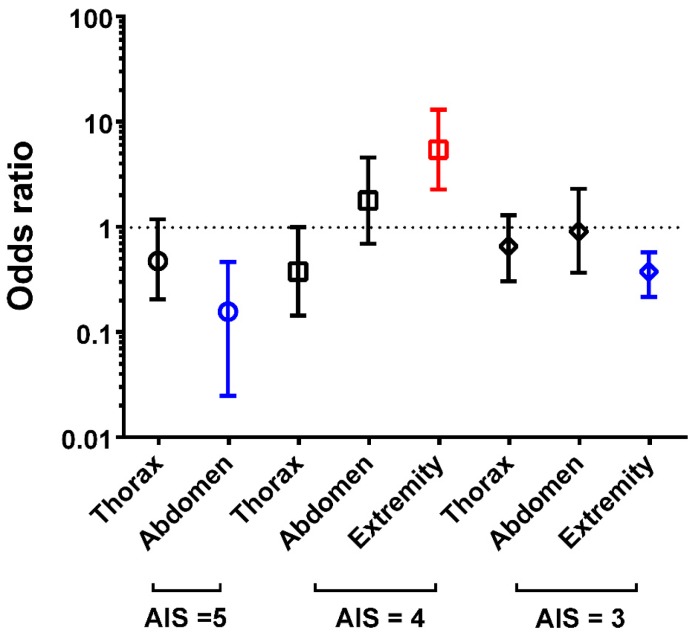
Odds of the adjusted mortality in the patients with injuries to thorax, abdomen, and extremity than the patients with injury to head/neck in different AIS stratum.

A summary of the odds of adjusted mortality in the patients with AIS of 3 to 5 in different regions is shown in Figure 2. The patients with abdomen injury of AIS of 5 and the patients with extremity injury of AIS of 3 had significantly lower odds of adjusted mortality than the patients with head/neck injury. However, the patients with extremity injury of AIS of 4 had significantly higher odds of adjusted mortality than the patients with head/neck injury.

This study compared the mortality outcomes of hospitalized trauma patients with the same AIS value of 3 to 5 in only one body region and found that the risks to mortality in the patients with a given AIS value in different injured body regions were not the same, even under the control of the confounding variables such as sex, age, comorbidities, and ISS. When compared to the patients with head/neck injury, significantly lower odds of adjusted mortality was noted in the patients with abdomen injury of AIS of 5 and the patients with extremity injury of AIS of 3, but significantly higher odds of adjusted mortality was noted in the patients with extremity injury of AIS of 4. The results contradicted the assumption that the patients with a given AIS value in different injured body regions would have a similar risk to mortality.

It had been reported that the imprecision of AIS was partly attributed to the expert-assigned severities of the AIS for each of the 1322 injuries in the AIS lexicon in the past and the calculation of empirical values was simply infeasible [9,10]. Because only six severity grades are available in the AIS, assigning the same severity may have altered the mortalities within a given AIS severity level across all body regions [9,10]. When only International Classification of Diseases (ICD)-9-CM codes are reported, ICD-9-CM-based severity scores perform even better than severity scores based on the conversion to AIS [11]. Furthermore, there was an overlap of the ranges of the mortality risk ratio value between different AIS severity levels such that similar mortality rates may be observed in some injuries of different AIS values [12]. In addition, the mechanism of injury such as penetrating or blunt trauma may significantly have an impact on the mortality rates of a given AIS value [13]. For an AIS of 4 or 5 in the head, penetrating trauma patients had a significantly higher mortality than blunt patients even under the controlling of age, sex, and other AIS categories [13]. For an AIS of 3 in the extremity and for an AIS of 1 in the external region, penetrating trauma patients had higher adjusted mortality in the regression model, albeit unadjusted mortality for blunt and penetrating patients was similar [13]. Therefore, the mechanism-based mortality may differ in specific AIS values by body region and may be attributed to certain ranges of the ISS [13].

It has been reported that certain ISS values exist for which patient mortality varies significantly depending on which AIS triplet combination of the trauma patients [14,15,16,17]. In particular, for an ISS of 25, mortality for the patients with AIS triplet of 5, 0, and 0 was higher than the patients with AIS triplet of 4, 3, and 0 [14,15], which also comprised the largest difference being 32% for an ISS of 25 [16]. Some authors had reported that ISS underestimates the relative consequences of the patients with orthopedic injury [18], penetrating injury to the abdomen [19], and vascular injury [20]. In this study, we demonstrated that the risks to mortality were not the same in the patients with a given AIS value in different injured body regions. Obviously, as stated in the introduction to the current version of the AIS of 2005, “The precise dimensions of severity have not been explicitly determined because these components change with time”, it is infeasible to compare the outcomes of the AIS for each of the more than one thousand injuries in the AIS lexicon and the measurement of extent to which they have influenced the severity assigned is not possible. However, despite its well-known flaws, ISS is still used as the “gold standard” to indicate the injury severity of the trauma patients in many trauma centers. Therefore, we suggested that, before a comparison being performed, a matched distribution of patients with injuries to different body regions between different groups of trauma patients should be acquired first to reduce the inherit bias of AIS-derived calculation of ISS.

This study had some limitations. First, the number of patients with the injuries to some body regions was limited and this may have caused a bias in outcome assessment. Second, the retrospective design of the study may have carried a selection bias, and under such condition, we could only rely on the assumption that a uniform management of these patients was given. Third, the patients declared dead at the accident scene or on hospital arrival were not included in the study and may have led to a selection bias [21,22]. In addition, long-term mortality was not evaluated in this study. Furthermore, we can only assume there were a uniform treatment to these studied patients in the condition of lacking information regarding pharmacological history and pharmacological treatments as well as the collaboration between the physician and the care manager, which can attribute to the outcomes of the patients [23], thus may result in bias in the outcome measurement. Finally, changes in the AIS version may have altered the measured outcome and subsequent predictive value of the ISS, with regard to specific dimensions over time [24].

## 4. Conclusions

This study found that the risks to mortality in the patients with a given AIS value of serious to critical injury in different injured body regions were not the same, even under the control of the confounding variables such as sex, age, comorbidities, and ISS.

## Figures and Tables

**Table 1:** Injury characteristics and outcomes of patients with AIS of 5.

Variables	Head/Neck *n* = 404		Thorax *n* = 11		Abdomen *n* = 17	
Sex						
Male, *n* (%)	265	(65.6)	8	(72.7)	12	(70.6)
Female, *n* (%)	139	(34.4)	3	(27.3)	5	(29.4)
Age (years)	57.1	±19.3	49.8	±18.4	37.4	±15.5
Co-morbidities						
DM, *n* (%)	63	(15.6)	1	(9.1)	1	(5.9)
HTN, *n* (%)	131	(32.4)	1	(9.1)	1	(5.9)
CAD, *n* (%)	22	(5.4)	0	(0.0)	0	(0.0)
CHF, *n* (%)	4	(1.0)	0	(0.0)	0	(0.0)
CVA, *n* (%)	22	(5.4)	0	(0.0)	0	(0.0)
ESRD, *n* (%)	17	(4.2)	0	(0.0)	0	(0.0)
Mechanisms						
Driver (motor vehicle), *n* (%)	4	(1.0)	0	(0.0)	3	(17.6)
Passenger (motor vehicle), *n* (%)	4	(1.0)	1	(9.1)	1	(5.9)
Driver (motorcycle), *n* (%)	165	(40.8)	3	(27.3)	10	(58.8)
Pillion (motorcycle), *n* (%)	10	(2.5)	1	(9.1)	1	(5.9)
Bicyclist, *n* (%)	27	(6.7)	0	(0.0)	0	(0.0)
Pedestrian, *n* (%)	17	(4.2)	0	(0.0)	1	(5.9)
Fall, *n* (%)	163	(40.3)	6	(54.5)	1	(5.9)
Struck by/against, *n* (%)	14	(3.5)	0	(0.0)	0	(0.0)
ISS	26.3	±2.2	26.4	±2.0	27.3	±2.3
Mortality, *n* (%)	179	(44.3)	1	(9.1)	1	(5.9)

CAD = coronary artery disease; CHF = congestive heart failure; CI = confidence interval; CVA = cerebral vascular accident; DM = diabetes mellitus; ESRD = end-stage renal disease; GCS = Glasgow coma scale; HTN = hypertension; ICU = intensive care unit; ISS = injury severity score; LOS = length of stay; OR = odds ratio.

**Table 2:** Injury characteristics and outcomes of patients with AIS of 5 (Patients with combined injuries).

Variables	Head/Neck *n* = 1680		Thorax *n* = 256		Abdomen *n* = 59		Extremity *n* = 22	
Sex								
Male, *n* (%)	339	(67.4)	15	(78.9)	19	(65.5)	2	(33.3)
Female, *n* (%)	164	(32.6)	4	(21.1)	10	(34.5)	4	(66.7)
Age (years)	55.5	±19.2	46.7	±19.5	39.1	±16.0	44.2	±12.8
Co-morbidities								
DM, *n* (%)	78	(15.5)	1	(5.3)	1	(3.4)	0	(0.0)
HTN, *n* (%)	154	(30.6)	1	(5.3)	1	(3.4)	1	(16.7)
CAD, *n* (%)	24	(5.4)	0	(0.0)	0	(0.0)	0	(0.0)
CHF, *n* (%)	4	(0.8)	0	(0.0)	0	(0.0)	0	(0.0)
CVA, *n* (%)	23	(4.6)	1	(5.3)	0	(0.0)	0	(0.0)
ESRD, *n* (%)	17	(3.4)	0	(0.0)	0	(0.0)	0	(0.0)
Mechanisms								
Driver (motor vehicle), *n* (%)	11	(2.2)	1	(5.3)	6	(20.7)	0	(0.0)
Passenger (motor vehicle), *n* (%)	6	(1.2)	2	(10.5)	1	(3.4)	0	(0.0)
Driver (motorcycle), *n* (%)	226	(44.9)	9	(47.4)	16	(55.2)	2	(33.3)
Pillion (motorcycle), *n* (%)	12	(2.4)	1	(5.3)	1	(3.4)	2	(33.3)
Bicyclist, *n* (%)	31	(6.2)	0	(0.0)	0	(0.0)	1	(16.7)
Pedestrian, *n* (%)	24	(4.8)	0	(0.0)	1	(3.4)	0	(0.0)
Fall, *n* (%)	177	(35.2)	6	(31.6)	3	(10.3)	1	(16.7)
Struck by/against, *n* (%)	16	(3.2)	0	(0.0)	1	(3.4)	0	(0.0)
ISS	29.1	±6.8	33.5	±10.5	33.4	±8.2	36.3	±12.7
Temperature (°C)	36.4	±1.8	36.3	±0.7	36.3	±0.4	36.2	±0.2
Heart rate (beats/min)	91.6	±26.7	99.5	±21.1	100.3	±20.0	98.3	±24.4
Respiratory rate (times/min)	19.0	±4.8	22.7	±9.2	21.3	±4.5	23.0	±8.5
Systolic blood pressure (mmHg)	155.9	±48.9	122.8	±30.5	103.4	±27.6	105.7	±36.9
AIS ≥ 3 in other region (s)	99	(19.7)	8	(42.1)	12	(41.4)	3	(50.0)
Mortality, *n* (%)	229	(45.5)	5	(26.3)	1	(3.4)	3	(50.0)

CAD = coronary artery disease; CHF = congestive heart failure; CVA = cerebral vascular accident; ESRD = end-stage renal disease; GCS = Glasgow coma scale; HTN = hypertension; ICU = intensive care unit; ISS = injury severity score; LOS = length of stay.

**Table 3:** Comparison of injury characteristics and outcomes of patients with injury to thorax, abdomen, and extremity vs. injury to head/neck in the patients with AIS 5 (Patients with combined injuries).

	Thorax vs. Head/Neck			Abdomen vs. Head/Neck		
Variables	Odds Ratio (95% CI)		*p*	Odds Ratio (95% CI)		*p*
Sex						
Male, *n* (%)	1.8	(0.59–5.55)	0.331	0.9	(0.42–2.02)	0.840
Female, *n* (%)	0.6	(0.18–1.69)	0.331	1.1	(0.50–2.39)	0.840
Age (years)	-		0.050	-		<0.001
Co-morbidities						
DM, *n* (%)	0.3	(0.04–2.30)	0.334	0.2	(0.03–1.45)	0.103
HTN, *n* (%)	0.1	(0.02–0.95)	0.019	0.1	(0.01–0.60)	0.003
CAD, *n* (%)	-		0.615	-		0.388
CHF, *n* (%)	-		1.000	-		1.000
CVA, *n* (%)	1.2	(0.15–9.07)	1.000	-		0.391
ESRD, *n* (%)	-		0.650	-		0.615
Mechanisms						
Driver (motor vehicle), *n* (%)	2.5	(0.30–20.30)	0.362	11.7	(3.97–34.33)	<0.001
Passenger (motor vehicle), *n* (%)	9.7	(1.83–51.86)	0.031	3.0	(0.34–25.42)	0.326
Driver (motorcycle), *n* (%)	1.1	(0.44–2.76)	1.000	1.5	(0.71–3.20)	0.339
Pillion (motorcycle), *n* (%)	2.3	(0.28–18.44)	0.386	1.5	(0.18–11.64)	1.000
Bicyclist, *n* (%)	-		0.402	-		0.247
Pedestrian, *n* (%)	-		0.618	0.7	(0.09–5.46)	1.000
Fall, *n* (%)	0.9	(0.32–2.28)	0.812	0.2	(0.06–0.71)	0.007
Struck by/against, *n* (%)	-		0.659	1.1	(0.14–8.49)	1.000
ISS	-		0.090	-		0.009
Temperature (°C)	-		0.696	-		0.678
Heart rate (beats/min)	-		0.204	-		0.085
Respiratory rate (times/min)	-		0.100	-		0.013
Systolic blood pressure (mmHg)	-		0.004	-		<0.001
AIS ≥ 3 in other region (s)	3.0	(1.16–7.57)	0.024	2.8	(1.33–6.23)	0.009
Mortality, *n* (%)	0.4	(0.15–1.20)	0.107	0.04	(0.01–0.32)	<0.001
Adjusted mortality	0.4	(0.14–1.25)	0.118	0.1	(0.01–0.39)	0.004
Log-Rank test	-		0.219	-		<0.001

CAD = coronary artery disease; CHF = congestive heart failure; CI = confidence interval; CVA = cerebral vascular accident; DM = diabetes mellitus; ESRD = end-stage renal disease; GCS = Glasgow coma scale; HTN = hypertension; ICU = intensive care unit; ISS = injury severity score; LOS = length of stay; OR = odds ratio.

**Table 4:** Injury characteristics and outcomes of patients with AIS of 4.

Variables	Head/Neck *n* = 1680		Thorax *n* = 256		Abdomen *n* = 59		Extremity *n* = 22	
Sex								
Male, *n* (%)	1077	(64.1)	191	(74.6)	37	(62.7)	12	(54.5)
Female, *n* (%)	603	(35.9)	65	(25.4)	22	(37.3)	10	(45.5)
Age (years)	58.0	±19.4	52.1	±15.2	40.9	±16.8	56.0	±19.3
Co-morbidities								
DM, *n* (%)	324	(19.3)	37	(14.5)	2	(3.4)	1	(4.5)
HTN, *n* (%)	591	(35.2)	60	(23.4)	5	(8.5)	2	(9.1)
CAD, *n* (%)	89	(5.3)	8	(3.1)	0	(0.0)	0	(0.0)
CHF, *n* (%)	14	(0.8)	0	(0.0)	0	(0.0)	0	(0.0)
CVA, *n* (%)	114	(6.8)	8	(3.1)	0	(0.0)	0	(0.0)
ESRD, *n* (%)	54	(3.2)	1	(0.4)	1	(1.7)	0	(0.0)
Mechanisms								
Driver (motor vehicle), *n* (%)	13	(0.8)	16	(6.2	11	(18.6)	0	(0.0)
Passenger (motor vehicle), *n* (%)	9	(0.5)	6	(2.3)	2	(3.4)	1	(4.5)
Driver (motorcycle), *n* (%)	749	(44.6)	157	(61.3)	31	(52.5)	9	(40.9)
Pillion (motorcycle), *n* (%)	28	(1.7)	3	(1.2)	0	(0.0)	0	(0.0)
Bicyclist, *n* (%)	90	(5.4)	3	(1.2)	2	(3.4)	3	(13.6)
Pedestrian, *n* (%)	67	(4.0)	9	(3.5)	1	(1.7)	0	(0.0)
Fall, *n* (%)	663	(39.5)	46	(18.0)	6	(10.2)	5	(22.7)
Struck by/against, *n* (%)	61	(3.6)	16	(6.2)	6	(10.2)	4	(18.2)
ISS	17.7	±2.5	19.0	±2.7	18.0	±2.4	17.7	±2.1
Temperature (°C)	36.5	±1.1	36.5	±0.7	36.4	±0.8	36.4	±1.1
Heart rate (beats/min)	86.2	±18.5	93.6	±18.8	89.9	±19.3	103.9	±25.2
Respiratory rate (times/min)	18.7	±2.6	19.2	±2.7	19.2	±2.6	20.8	±4.8
Systolic blood pressure (mmHg)	156.6	±35.3	141.9	±33.4	118.6	±31.6	101.8	±42.7
Mortality, *n* (%)	80	(4.8)	3	(1.2)	4	(6.8)	5	(22.7)

CAD = coronary artery disease; CHF = congestive heart failure; CVA = cerebral vascular accident; ESRD = end-stage renal disease; GCS = Glasgow coma scale; HTN = hypertension; ICU = intensive care unit; ISS = injury severity score; LOS = length of stay.

**Table 5:** Comparison of injury characteristics and outcomes of patients with injury to thorax, abdomen, and extremity vs. injury to head/neck in the patients with AIS 4.

	Thorax vs. Head/Neck			Abdomen vs. Head/Neck			Extremity vs. Head/Neck		
Variables	Odds Ratio (95% CI)		*p*	Odds Ratio (95% CI)		*p*	Odds Ratio (95% CI)		*p*
Sex									
Male, *n* (%)	1.6	(1.22–2.22)	0.001	0.9	(0.55–1.61)	0.890	0.7	(0.29–1.56)	0.376
Female, *n* (%)	0.6	(0.45–0.82)	0.001	1.1	(0.62–1.82)	0.890	1.5	(0.64–3.47)	0.376
Age (years)	-		<0.001	-		<0.001	-		0.622
Co-morbidities									
DM, *n* (%)	0.7	(0.49–1.02)	0.070	0.1	(0.04–0.61)	0.002	0.2	(0.03–1.49)	0.100
HTN, *n* (%)	0.6	(0.42–0.77)	<0.001	0.2	(0.07–0.43)	<0.001	0.2	(0.04–0.79)	0.011
CAD, *n* (%)	0.6	(0.28–1.20)	0.166	-		0.071	-		0.408
CHF, *n* (%)	-		0.238	-		1.000	-		1.000
CVA, *n* (%)	0.4	(0.21–0.92)	0.026	-		0.052	-		0.394
ESRD, *n* (%)	0.1	(0.02–0.86)	0.013	0.5	(0.07–3.82)	0.721	-		0.642
Mechanisms									
Driver (motor vehicle), *n* (%)	8.5	(4.06–17.99)	<0.001	29.4	(12.53–68.94)	<0.001	-		1.000
Passenger (motor vehicle), *n* (%)	4.5	(1.57–12.63)	0.009	6.5	(1.38–30.84)	0.051	8.8	(1.07–72.95)	0.122
Driver (motorcycle), *n* (%)	2.0	(1.51–2.58)	<0.001	1.4	(0.82–2.32)	0.234	0.9	(0.37–2.02)	0.831
Pillion (motorcycle), *n* (%)	0.7	(0.21–2.32)	0.616	-		0.623	-		1.000
Bicyclist, *n* (%)	0.2	(0.07–0.67)	0.004	0.6	(0.15–2.58)	0.584	2.8	(0.81–9.60)	0.115
Pedestrian, *n* (%)	0.9	(0.43–1.78)	0.738	0.4	(0.06–3.04)	0.515	-		0.625
Fall, *n* (%)	0.3	(0.24–0.47)	<0.001	0.2	(0.07–0.41)	<0.001	0.5	(0.17–1.23)	0.127
Struck by/against, *n* (%)	1.8	(1.00–3.12)	0.057	3.0	(1.24–7.26)	0.024	5.9	(1.94–17.95)	0.008
ISS	-		<0.001	-		0.451	-		0.991
Temperature (°C)	-		0.306	-		0.728	-		0.839
Heart rate (beats/min)	-		<0.001	-		0.131	-		0.004
Respiratory rate (times/min)	-		0.004	-		0.158	-		0.053
Systolic blood pressure (mmHg)	-		<0.001	-		<0.001	-		<0.001
Mortality, *n* (%)	0.2	(0.07–0.76)	0.012	1.5	(0.51–4.11)	0.528	5.9	(2.12–16.35)	0.004
Adjusted mortality	0.3	(0.09–1.01)	0.051	2.1	(0.64–6.80)	0.220	8.4	(2.84–25.07)	<0.001
Log-Rank test	-		0.003	-		0.713	-		0.028

CAD = coronary artery disease; CHF = Congestive Heart Failure; CI = confidence interval; CVA = cerebral vascular accident; DM = diabetes mellitus; ESRD = end-stage renal disease; GCS = Glasgow coma scale; HTN = hypertension; ICU = intensive care unit; ISS = injury severity score; LOS = length of stay; OR = odds ratio.

**Table 6:** Injury characteristics and outcomes of patients with AIS of 3.

Variables	Head/Neck *n* = 1178		Thorax *n* = 749		Abdomen *n* = 253		Extremity *n* = 5967	
Sex								
Male, *n* (%)	686	(58.2)	517	(69.0)	151	(59.7)	2599	(43.6)
Female, *n* (%)	492	(41.8)	232	(31.0)	102	(40.3)	3368	(56.4)
Age (years)	54.2	±18.7	54.6	±16.1	48.5	±18.0	62.2	±19.9
Co-morbidities								
DM, *n* (%)	177	(15.0)	119	(15.9)	34	(13.4)	1373	(23.0)
HTN, *n* (%)	371	(31.5)	211	(28.2)	55	(21.7)	2519	(42.4)
CAD, *n* (%)	36	(3.1)	20	(2.7)	6	(2.4)	379	(6.4)
CHF, *n* (%)	12	(1.0)	4	(0.5)	2	(0.8)	100	(1.7)
CVA, *n* (%)	59	(5.0)	17	(2.3)	6	(2.4)	490	(8.2)
ESRD, *n* (%)	20	(1.7)	10	(1.3)	1	(0.4)	191	(3.2)
Mechanisms								
Driver (motor vehicle), *n* (%)	21	(1.8)	31	(4.1)	18	(7.1)	56	(0.9)
Passenger (motor vehicle), *n* (%)	14	(1.2)	12	(1.6)	6	(2.4)	28	(0.5)
Driver (motorcycle), *n* (%)	650	(55.2)	432	(57.7)	99	(39.1)	1848	(31.0)
Pillion (motorcycle), *n* (%)	31	(2.6)	13	(1.7)	4	(1.6)	125	(2.1)
Bicyclist, *n* (%)	63	(5.3)	23	(3.1)	3	(1.2)	232	(3.9)
Pedestrian, *n* (%)	37	(3.1)	12	(1.6)	4	(1.6)	115	(1.9)
Fall, *n* (%)	310	(26.3)	173	(23.1)	92	(36.4)	3317	(55.6)
Struck by/against, *n* (%)	52	(4.4)	53	(7.1)	27	(10.7)	246	(4.1)
ISS	11.0	±2.4	11.7	±2.5	10.4	±2.0	9.3	±1.1
Temperature (°C)	36.5	±1.1	36.5	±0.7	36.4	±0.8	36.4	±1.1
Heart rate (beats/min)	86.2	±18.5	93.6	±18.8	89.9	±19.3	103.9	±25.2
Respiratory rate (times/min)	18.7	±2.6	19.2	±2.7	19.2	±2.6	20.8	±4.8
Systolic blood pressure (mmHg)	156.6	±35.3	141.9	±33.4	118.6	±31.6	101.8	±42.7
Mortality, *n* (%)	19	(1.6)	7	(0.9)	4	(1.6)	47	(0.8)

CAD = coronary artery disease; CHF = congestive heart failure; CVA = cerebral vascular accident; ESRD = end-stage renal disease; GCS = Glasgow coma scale; HTN = hypertension; ICU = intensive care unit; ISS = injury severity score; LOS = length of stay.

**Table 7:** Comparison of injury characteristics and outcomes of patients with injury to thorax, abdomen, and extremity vs. injury to head/neck in the patients with AIS 3.

	Thorax vs. Head/Neck			Abdomen vs. Head/Neck			Extremity vs. Head/Neck		
Variables	Odds Ratio (95% CI)		*p*	Odds Ratio (95% CI)		*p*	Odds Ratio (95% CI)		*p*
Sex									
Male, *n* (%)	1.6	(1.32–1.94)	<0.001	1.1	(0.81–1.40)	0.674	0.6	(0.49–0.63)	<0.001
Female, *n* (%)	0.6	(0.52–0.76)	<0.001	0.9	(0.71–1.24)	0.674	1.8	(1.59–2.05)	<0.001
Age (years)	-		0.625	-		<0.001	-		<0.001
Co-morbidities									
DM, *n* (%)	1.1	(0.83–1.38)	0.650	0.9	(0.59–1.30)	0.559	1.7	(1.43–2.01)	<0.001
HTN, *n* (%)	0.9	(0.70–1.04)	0.127	0.6	(0.44–0.84)	0.002	1.6	(1.39–1.82)	<0.001
CAD, *n* (%)	0.9	(0.50–1.52)	0.678	0.8	(0.32–1.85)	0.684	2.2	(1.52–3.05)	<0.001
CHF, *n* (%)	0.5	(0.17–1.62)	0.310	0.8	(0.17–3.48)	1.000	1.7	(0.91–3.02)	0.122
CVA, *n* (%)	0.4	(0.26–0.76)	0.003	0.5	(0.20–1.08)	0.094	1.7	(1.29–2.24)	<0.001
ESRD, *n* (%)	0.8	(0.37–1.68)	0.577	0.2	(0.03–1.72)	0.153	1.9	(1.20–3.05)	0.006
Mechanisms									
Driver (motor vehicle), *n* (%)	2.4	(1.36–4.17)	0.002	4.2	(2.21–8.04)	<0.001	0.5	(0.32–0.87)	0.014
Passenger (motor vehicle), *n* (%)	1.4	(0.62–2.94)	0.544	2.0	(0.77–5.31)	0.232	0.4	(0.21–0.75)	0.006
Driver (motorcycle), *n* (%)	1.1	(0.92–1.33)	0.300	0.5	(0.40–0.69)	<0.001	0.4	(0.32–0.41)	<0.001
Pillion (motorcycle), *n* (%)	0.7	(0.34–1.26)	0.215	0.6	(0.21–1.70)	0.381	0.8	(0.53–1.18)	0.274
Bicyclist, *n* (%)	0.6	(0.35–0.91)	0.023	0.2	(0.07–0.68)	0.004	0.7	(0.54–0.95)	0.025
Pedestrian, *n* (%)	0.5	(0.26–0.97)	0.038	0.5	(0.18–1.40)	0.216	0.6	(0.42–0.88)	0.011
Fall, *n* (%)	0.8	(0.68–1.04)	0.118	1.6	(1.20–2.13)	0.002	3.5	(3.05–4.03)	<0.001
Struck by/against, *n* (%)	1.6	(1.11–2.45)	0.013	2.6	(1.59–4.21)	<0.001	0.9	(0.69–1.26)	0.690
ISS	-		<0.001	-		<0.001	-		<0.001
Temperature (°C)	-		0.710	-		0.918	-		0.103
Heart rate (beats/min)	-		0.377	-		0.055	-		0.573
Respiratory rate (times/min)	-		0.699	-		0.330	-		0.074
Systolic blood pressure (mmHg)	-		0.038	-		<0.001	-		0.499
Mortality, *n* (%)	0.6	(0.24–1.38)	0.231	1.0	(0.33–2.91)	1.000	0.5	(0.28–0.83)	0.009
Adjusted mortality	0.6	(0.23–1.40)	0.218	0.9	(0.29–2.89)	0.875	0.3	(0.15–0.51)	<0.001
Log-Rank test	-		0.100	-		0.719	-		0.001

CAD = coronary artery disease; CHF = congestive heart failure; CI = confidence interval; CVA = cerebral vascular accident; DM = diabetes mellitus; ESRD = end-stage renal disease; GCS = Glasgow coma scale; HTN = hypertension; ICU = intensive care unit; ISS = injury severity score; LOS = length of stay; OR = odds ratio.
